# Autophagy-enhancing strategies to promote intestinal viral resistance and mucosal barrier function in SARS-CoV-2 infection

**DOI:** 10.1080/27694127.2025.2514232

**Published:** 2025-06-10

**Authors:** Anusca G. Rader, Alexandra P.M. Cloherty, Kharishma S. Patel, Dima D.A. Almandawi, Jimena Perez-Vargas, Manon E. Wildenberg, Vanesa Muncan, Renée R.C.E. Schreurs, François Jean, Carla M.S. Ribeiro

**Affiliations:** a Center for Infection and Molecular Medicine, Amsterdam UMC Location University of Amsterdam, Amsterdam, The Netherlands; b Experimental Immunology, Amsterdam UMC Location University of Amsterdam, Amsterdam, The Netherlands; c Amsterdam Institute for Immunology & Infectious Diseases, Amsterdam, The Netherlands; d Amsterdam Gastroenterology Endocrinology and Metabolism, Amsterdam, The Netherlands; e Department of Microbiology and Immunology, Life Sciences Institute, University of British Columbia, Vancouver, British Columbia, Canada; f Amsterdam Gastroenterology and Hepatology, Tytgat Institute for Liver and Intestinal Research, Amsterdam UMC Location University of Amsterdam, Amsterdam, The Netherlands

**Keywords:** Antiviral immunity, autophagy, epithelial cells, human intestinal organoids, host-directed therapy, SARS-CoV-2, gastrointestinal pathology, post-COVID-19 condition

## Abstract

Severe acute respiratory syndrome coronavirus 2 (SARS-CoV-2), the causative agent of Coronavirus disease 19 (COVID-19), continues to circulate globally despite the widespread vaccination and therapeutics like Paxlovid, remdesivir, and molnupiravir. COVID-19 is associated with both respiratory and gastrointestinal manifestations, with persistent intestinal pathology contributing to the post-COVID-19 condition. We have previously demonstrated the antiviral activity of autophagy-blocking drugs, such as Berbamine dihydrochloride, against intestinal SARS-CoV-2 acquisition. In addition, the autophagy blockers restored the barrier function of infected intestinal epithelium. In this addendum, using human intestinal organoids, we present evidence for a protective role of intrinsic higher levels of autophagy flux in limiting intestinal SARS-CoV-2 infection. Pharmacological treatment with Akt inhibitor MK-2206 hydrochloride suppressed viral entry into the intestinal epithelium. This antiviral effect of MK-2206 was shown to be dependent on Synaptosomal-associated protein 29-dependent (SNAP-29)-mediated autophagy flux. Furthermore, extrinsically enhanced autophagy with MK-2206 also prevented SARS-CoV-2-induced intestinal barrier damage. Our findings thus underscore the intricate role of autophagy pathways in the dissemination and pathogenesis of intestinal SARS-CoV-2, highlighting the therapeutic potential of host-directed therapies targeting autophagy to intervene in COVID-19-associated sequelae and improve intestinal health.

## Introduction

Since the emergence of severe acute respiratory syndrome coronavirus 2 (SARS-CoV-2) in December 2019, the COVID-19 pandemic has led to more than 760 million recorded cases and over 7 million deaths worldwide [1,2]. While SARS-CoV-2 is no longer classified as a public health emergency, the virus continues to circulate globally. In addition, individuals with acute SARS-CoV-2 infection are at risk of developing persistent or relapsing multisystem symptoms collectively defined as the post-COVID-19 condition [3]. The most common symptoms include fatigue, shortness of breath, and cognitive impairment. To date, the pathophysiological mechanisms underlying the post-COVID-19 condition remain elusive, and no curative treatment has been identified [3,4]. Although SARS-CoV-2 is primarily recognized as a respiratory virus, it is evident that extrapulmonary organs are also affected. In particular, gastrointestinal dysfunction has been implicated in both acute COVID-19 infections and the post-COVID-19 condition [5–7]. The broad expression of host SARS-CoV-2 receptor ACE2 (angiotensin-converting enzyme 2) along the gut lining promotes intestinal viral infection, which subsequently leads to disruption of intestinal epithelial barrier function and triggers mucosal inflammation. These effects contribute to gastrointestinal symptoms during acute COVID-19, such as diarrhea, nausea, and abdominal pain [5,7–9]. Furthermore, increased gut permeability, persistence of SARS-CoV-2 antigens, and gut microbiome dysbiosis support the translocation of microbial components and inflammatory mediators into the bloodstream, which has been associated with systemic inflammation and prolonged gastrointestinal manifestations in COVID-19 pathogenesis [5,6,10]. Over the past five years, intensive efforts have been made to elucidate the complex interplay between multiorgan dissemination of SARS-CoV-2 and host immune response mechanisms, aiming to identify therapeutic targets for COVID-19 disease [11–14]. Notably, host autophagy has emerged as a key player in viral acquisition and disease severity [15–19]. In addition to the cell-surface routing [20,21], we and others have previously confirmed a proviral role of autophagy machinery in the endosomal entry route of SARS-CoV-2 [18,22]. This entry mechanism relies on an acidic intravesicular environment that facilitates the fusion of viral and endosomal membranes for subsequent release of the coronaviral genome into the cell and establishment of infection [21]. In our previous study [18], we demonstrated that the autophagy-blocking therapeutic Berbamine dihydrochloride robustly prevents SARS-CoV-2 acquisition by human intestinal epithelial cells via an autophagy-mediated Bcl-2 interacting protein 3 (BNIP3) mechanism, which underscores the remedial potential of host-directed strategies targeting autophagosome-lysosome fusion to intervene in gastrointestinal COVID-19 [18]. Notably, autophagy appears to play a dichotomous role during SARS-CoV-2 infection. On one hand, SARS-CoV-2 exploits acidified autophagic vesicles, as a result of autophagosome-lysosome fusion, as an endosomal entry route to establish infection in pulmonary and intestinal epithelial cells [18,23]. On the other hand, SARS-CoV-2 has been shown to induce autophagosome formation while concurrently blocking autophagy flux, thereby preventing degradation of viral components and supporting its replication cycle in susceptible cell lines, including those of pulmonary and intestinal origin [24–26]. Furthermore, previous studies have identified beneficial effects of autophagy-inducing compounds in limiting SARS-CoV-2 infection, further underscoring the relevance of functional autophagy flux to promote viral resistance [27,28]. In this addendum to Cloherty & Rader et al. (2023) [18], we expand our studies on autophagy-based antivirals for enteric SARS-CoV-2 by investigating the impact of enhancement of autophagy flux on intestinal SARS-CoV-2 infection and associated intestinal dysfunction.

## Results and discussion

### Intrinsically higher levels of autophagy in genotyped intestinal epithelial cells are associated with decreased susceptibility to SARS-CoV-2 infection

**Figure 1.**
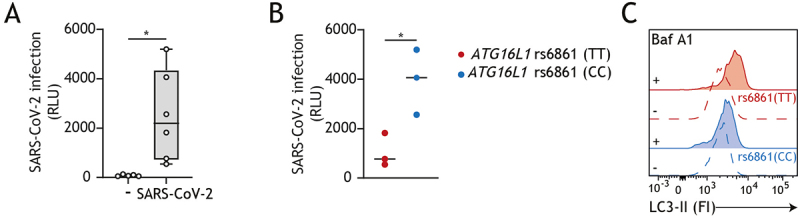
Intrinsic higher number of autophagosomes correlates with reduced intestinal SARS-CoV-2 infection. (A,B) viral infection of confluent intestinal epithelial monolayers exposed to SARS-CoV-2 pseudovirus for 5 days, determined by luciferase activity (relative light units, RLU). (A) Data are mean ± SD of *n *= 6 donors represented by open circles; *p < 0.05. (B) Stratification of viral infection levels in genotyped intestines: *ATG16L1* rs6861(TT) donors (red colored circles) versus *ATG16L1* rs6861 (CC) donors (blue-colored circles); *p < 0.05. (C) Flow-cytometric representation of autophagy flux as measured by saponin extraction of intracellular LC3-II accumulation (in fluorescent intensity, FI) upon incubation with 100 nM bafilomycin A1 for 4 h in *ATG16L1* rs6861 (TT, red, versus CC, blue) genotyped intestinal epithelial cells. Data are representative of *n*=2 donors per genotype.

SARS-CoV-2 infection displays a variable clinical presentation that ranges from asymptomatic infection to critical illness. Besides the general risk factors such as advanced age, male sex and chronic comorbidities, convergent studies provided evidence that host genetics may contribute to the development of COVID-19 severity [29–31]. Recently, we have identified a naturally occurring single nucleotide polymorphism (SNP) in the core autophagy gene *ATG16L1* (Autophagy-related 16 like 1), essential for autophagosome formation, which is clinically associated with delayed disease progression and improved survival in chronic HIV-1 infection [32]. We have shown that carriers of the newly identified *ATG16L1* rs6861 (TT) genotype exhibit intrinsically enhanced autophagy flux, which correlates with improved T cell-mediated antiviral immunity and superior immunoregulatory capacity in chronically HIV-1-infected individuals [32]. In our studies on intestinal SARS-CoV-2 infection, we have observed a noteworthy spread in the rate of infection across the different gut donors (Figure 1A). Further stratification of these donors based on their genotype revealed that the SARS-CoV-2 infection levels in the intestinal epithelium were relatively lower in *ATG16L1* rs6861(TT) genotyped donors as compared to *ATG16L1* rs6861 (CC) (Figure 1B). Moreover, consistent with the enhanced autophagic activity reported for genotyped primary T-cells and dendritic cells [32,33], subsequent analyses of autophagy flux confirmed that the *ATG16L1* rs6861 (TT) genotyped intestinal epithelial cells displayed higher number of autophagosomes as compared to rs6861(CC) genotype, as shown by increased intracellular LC3-II levels following bafilomycin A1 treatment (Figure 1C). Notably, genetic variations in key autophagy genes have been associated with the onset or severity of a range of human diseases. For example, the A*TG16L1* rs2241880 (T300A) variant increases the predisposition to inflammatory bowel disease and has been associated with a higher risk of hepatitis B virus infection [34–36]. Additional autophagy-related polymorphisms, such as *IRGM (Immunity-Related GTPase M)* rs13361189 and *ATG7 (Autophagy-related 7)* rs36117895, have been linked to neurological, inflammatory, degenerative disorders, and cancer [37]. In the context of SARS-CoV-2, a recent study reported differences in the frequencies of the *ATG16L1* rs2241880 and *ATG5 (Autophagy-related 5)* rs506027 polymorphisms between COVID-19 patients and healthy controls [38]. Although we cannot infer causality between autophagy activity and SARS-CoV-2 infection rates in these genotyped intestinal donors, our findings suggest the relevance of a functional autophagic flux in thwarting intestinal SARS-CoV-2 dissemination and warrant further investigation into the clinical relevance of host genetic variation in autophagy machinery in COVID-19 pathogenesis.

### Autophagy-enhancing drug MK-2206 suppresses intestinal SARS-CoV-2 infection

**Figure 2.**
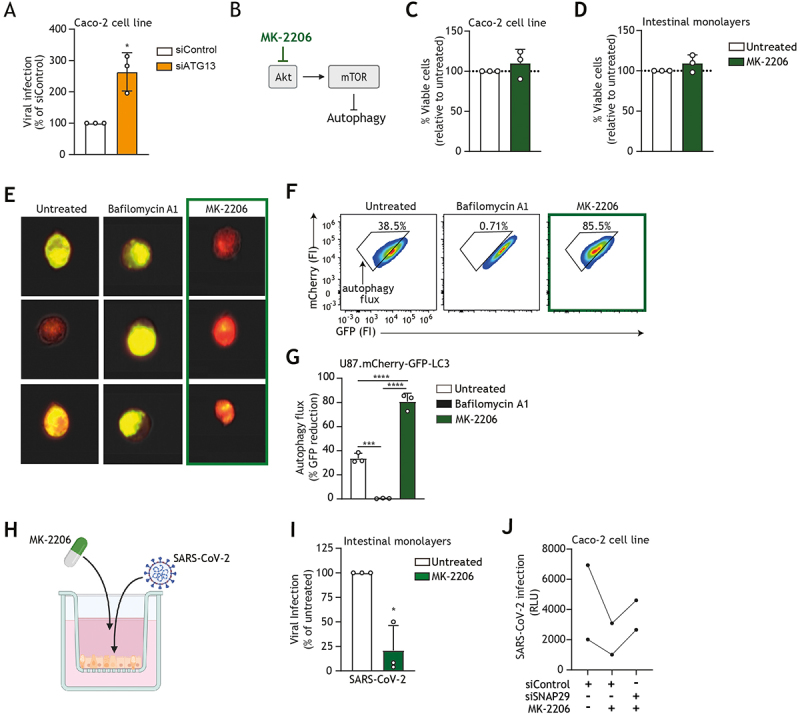
Therapeutic enhancement of autophagy by MK-2206 suppresses intestinal SARS-CoV-2 infection via SNAP29-mediated autophagic degradation. (A) Viral infection of Caco-2 cells upon transfection with control siRNA or siATG13, followed by exposure to pseudotyped SARS-CoV-2 for 72 h, determined by luciferase activity (RLU). Open circles represent individual replicates, *n* = 3; *p < 0.05. (B) Schematic representation of the mechanism by which Akt inhibitor MK-2206 impacts its molecular target to pharmaceutically increase autophagy flux. (C,D) Percentage cell viability upon treatment of Caco-2 cell line (C) or *ATG16L1* rs6861 (CC) genotyped intestinal epithelial monolayers (D) with MK-2206, determined by ATP-based CellTiter-Glo assay. Cells were treated with an optimized concentration of 5 μM MK-2206 or left untreated for 72 h (C) or 60 h (D); open circles represent individual replicates, *n* = 3. (E–G) Autophagy flux in U87.mCherry-GFP-LC3 autophagy reporter cell line upon incubation with 5 μM MK-2206 or 200 nM bafilomycin A1 as control, or left untreated for 24 or 48 h. Percentage GFP signal reduction is representative of autophagic flux, determined by imaging flow cytometry after 24 h (E) and flow cytometry after 48 h (F, G). (E) Representative imaging flow cytometry overlays for brightfield, mCherry, and GFP signals, three individual cells shown per condition are representative of *n* = 3 replicates (F) Representative flow cytometry plots, and (G) the quantification, data are mean ± SD of *n *= 3 replicates represented by open circles; ****p < 0.0001, ***p < 0.001. (H) Schematic representation of intestinal epithelial monolayer pre-treatment with MK-2206 and subsequent infection with SARS-CoV-2 pseudovirus. (I) Viral infection of confluent *ATG16L1* rs6861 (CC) genotyped intestinal epithelial monolayers pre-treated with 5 μM MK-2206 or left untreated for 24 h, followed by infection with SARS-CoV-2 pseudovirus for 5 days, determined by luciferase activity (RLU). Data were normalized to untreated and are mean ± SD of *n* = 3 donors represented by open circles; *p < 0.05. (J) Viral infection of Caco-2 cells upon transfection with control siRNA or si*SNAP29*, followed by treatment with 5 μM MK-2206 and subsequently exposed to pseudotyped SARS-CoV-2 for 72 h, determined by luciferase activity (RLU). Circles represent individual replicates, *n* = 2 replicates.

To investigate the role of autophagy initiation on SARS-CoV-2 entry of epithelial cells, we employed RNA interference (RNAi) technology in human colorectal Caco-2 cell line to knock down the expression of autophagy molecule ATG13 (autophagy-related 13), a molecule essential for autophagosome formation [39]. Notably, the knockdown of *ATG13* resulted in increased SARS-CoV-2 infection, underscoring the relevanceof ULK1-dependent autophagy pathway to prevent SARS-CoV-2 infection (Figure 2A, Fig. S1A). To further explore the beneficial effect of autophagy on the antiviral function of intestinal epithelium, we investigated the therapeutic potential of autophagy enhancement to promote viral resistance of human intestinal epithelium. To this end, we selected MK-2206 hydrochloride (hereafter MK-2206), a selective allosteric Akt inhibitor with known ability to enhance autophagy activity (Figure 2B,[28,32]), and currently under clinical trial for several cancer types [40–42]. Non-toxic concentrations of MK-2206 were established in both the Caco-2 cell line and primary human intestinal monolayers by an ATP-based cell viability assay (Figure 2C,D and Fig. S1D). Next, the capacity of MK-2206 to regulate autophagy flux was assessed in an engineered U87 human cell line using both imaging flow cytometry and flow cytometry. In the well-established U87.mCherry-GFP-LC3 autophagy reporter system, the LC3 protein is tandem fluorescent-tagged with both GFP and mCherry and the acid-sensitive GFP tag is quenched upon autophagosome-lysosome fusion. A reduction in GFP fluorescence thereby indicates enhanced autophagic flux, whereas a block of autophagy flux, as observed with bafilomycin A1 treatment, results in increased double-positive GFP+mCherry+ puncta, detected as discrete yellow fluorescence [43]. Treatment with MK-2206 strongly enhanced autophagy flux as determined by a marked reduction in GFP signal (Figure 2E–G). Next, human intestinal epithelial monolayers or Caco-2 cells were pre-treated with MK-2206 and subsequently infected with SARS-CoV-2 (Figure 2H). MK-2206 treatment suppressed SARS-CoV-2 entry into both human intestinal epithelium (Figure 2I) and Caco-2 cells (Fig. S1E). Additional autophagy-enhancing drugs such as everolimus or sirolimus (rapamycin), targeting mTOR complex [44], also resulted in a similar reduction of SARS-CoV-2 viral infection (Fig. S1C,F).

Next, we set out to investigate the mechanism underlying the MK-2206-mediated antiviral effect in the human intestinal epithelium. To this end, the expression of SNAP29, a key host molecule required for autophagosome – lysosome fusion, was silenced in Caco-2 cells using RNAi technology, after which cells were treated with MK-2206 and subsequently infected with SARS-CoV-2. In Caco-2 cells transfected with control siRNA, there remained a strong preventative effect of MK-2206 on SARS-CoV-2 acquisition (Figure 2J). Markedly, this antiviral effect was reduced upon the knockdown of SNAP29 (Figure 2J and Fig. S1B), suggesting that MK-2206 limits intestinal SARS-CoV-2 infection via a SNAP-29-mediated autophagic degradation. Together, these findings underscore the suitability of pharmaceutical enhancement of autophagy flux to enhance the antiviral function of human epithelial cells [28,32,40]. Given the documented *in vivo* beneficial effect of MK 2206 in advanced clinical trial phases for different cancer types [41,42] and its promising *in vitro* efficacy against viruses [28], MK-2206 holds potential as a pertinent therapeutic option for accelerated drug repurposing as an autophagy-based antiviral.

### SARS-CoV-2-induced barrier dysfunction is effectively alleviated by MK-2206 treatment

**Figure 3.**
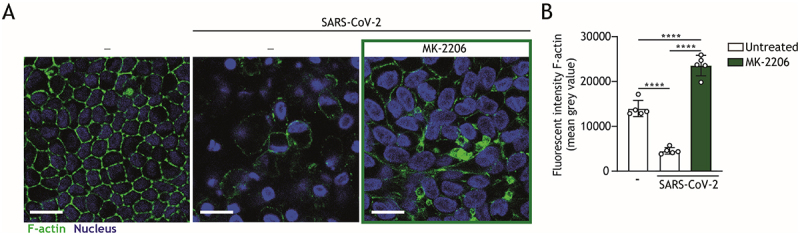
MK-2206 treatment hampers SARS-CoV-2-mediated disruption of the intestinal barrier. (A) Representative confocal microscopy imaging of the changes in morphology of *ATG16L1* rs6861 (CC) genotyped intestinal epithelial monolayers upon pre-treatment with 5 μM MK-2206 or left untreated for 24 h, followed by exposure to SARS-CoV-2 pseudovirus for 5 days. Actin (Phalloidin) is visualized in green and nuclei (DAPI) in blue. Scale bar = 15 μm, images representative of *n *= 2 donors. (B) Quantitative analysis of fluorescence intensity of actin staining, as depicted in (A). Fluorescent intensity was determined at the original magnification by measuring the mean gray value with ImageJ software. Data are mean ± SD of 5 fields of view of a representative donor, represented by open circles, *n *= 2 donors; ***p < 0.0001.

Autophagy is crucial for maintaining intestinal epithelial barrier integrity and promoting intestinal regeneration [45,46]. Disruptions in autophagic flux impair epithelial function as well as contribute to inflammatory gut disorders and compromised barrier function [47–49], emphasizing its essential role in intestinal health. COVID-19 has been associated with gastrointestinal pathogenesis and intestinal barrier permeability [18,50]. We therefore assessed whether the autophagy-inducing drug MK-2206 could block SARS-CoV-2-mediated damage to the intestinal barrier. Using immunofluorescent staining for cytoskeletal actin and nuclei, we observed that the SARS-CoV-2-induced loss of cell–cell contact, indicative of impaired barrier integrity, was largely prevented by treatment with the Akt inhibitor MK-2206 (Figure 3A,B).

Taken together, our findings demonstrate that both intrinsic and pharmaceutical enhancement of autophagy flux correlates with superior intestinal viral resistance. In line with our previous data showing that autophagy inhibitors suppress SARS-CoV-2 infection of intestinal epithelial cells [18], our current study further underscores the intricate bidirectional interplay between SARS-CoV-2 fusogenic entry and host autophagy. Our studies suggest that while autophagy-blocking therapeutics (such as Berbamine dihydrochloride) limit intestinal SARS-CoV-2 infection by preventing BNIP3-dependent endosomal viral entry route; autophagy-enhancing therapies (such as MK-2206) alternatively promote targeting of virions or viral components for autophagy-mediated SNAP29 lysosomal degradation, thereby preventing subsequent epithelial SARS-CoV-2 entry. Given the pivotal role of autophagy in intestinal stem cell homeostasis and mucosal immunity [44–47], further investigation into the mechanism of actions and therapeutic strategies with repurposable autophagy-modulating drugs, as demonstrated in this and our previous study [18], could mitigate long-lasting gastrointestinal manifestations in post-COVID-19 condition [49,50] and expand our toolkit for pandemic preparedness research with host-directed antivirals targeting autophagy.

## Material and methods

### Ethics statement

This study has been conducted in accordance with the ethical principles set out in the declaration of Helsinki. Human fetal intestinal tissue samples (gestational age 16–19 weeks) were obtained from the HIS Mouse Facility of the Amsterdam UMC, The Netherlands. All material has been collected from donors from whom a written informed consent has been obtained for the use of the material for research purposes. The fetal donor information is anonymized and is not available to the Amsterdam UMC. The use of the anonymized material for medical research purposes is covered by a Dutch law (Wet foetaal weefsel and Article 467 of Behandelingsovereenkomst). Tissues were obtained with approval of the experimental procedures by the HIS Mouse Facility (Amsterdam UMC).

### Genotyping

Genotyping to determine the *ATG16L1* rs6861 (TT and CC) genetic variants in human intestinal donors was performed on fetal intestinal tissues using the TaqMan Sample-to-SNP kit (ThermoFisher) according to manufacturer’s instructions [32].

### Intestinal epithelial monolayers

Human fetal intestinal organoids were generated from the human fetal intestine as described previously and were subsequently used to generate human intestinal epithelial monolayers [18,51]. Briefly, 3.0 µm pore 24-well transwell inserts (Corning) were coated with 20 µg/mL rat collagen type 1 (Ibidi) in 0.1% acetic acid (Sigma Aldrich). Single cells were obtained from gut organoids using TrypLE (Gibco, Thermo Fischer Scientific) digestion and suspended in expansion medium – Intesticult human organoid growth medium (OGM; STEMCELL Technologies) components “Human Basal Medium” and “Organoid Supplement” in a 1:1 ratio supplemented with penicillin/streptomycin (10 U/mL and 10 μg/mL, respectively; Thermo Fisher Scientific) – supplemented with Y-27632 (STEMCELL Technologies), followed by seeding onto the collagen-coated transwell inserts. For the first 7 days the monolayers were cultured in expansion medium. From day 7 onwards all monolayers were cultured in a differentiation medium (1:1 mixture of OGM component “Human Basal Medium”) and Advanced DMEM/F12 (Thermo Fisher Scientific) supplemented with 10 mm GlutaMAX (Thermo Fisher Scientific), 10 mm HEPES (Sigma), and penicillin/streptomycin (10 U/mL and 10 μg/mL, respectively; Invitrogen), and refreshed every 3–4 days. Epithelial monolayer integrity was routinely assessed, as previously described [18,52], by measuring trans-epithelial electrical resistance (TEER) using a voltohmmeter (EVOM2), with monolayers deemed confluent when TEER exceeded 200 Ω·cm^2^. TEER assays confirmed the establishment of an epithelial monolayer with tight barrier function before virus exposure [18,51].

### Intracellular LC3-II staining

Quantification of intracellular LC3-II levels in *ATG16L1* rs6861 genotyped intestinal epithelial organoids by saponin extraction was performed as described before [33]. Intestinal epithelial organoids were either left untreated or pre-treated with 100 nM bafilomycin A1 (Invivogen tlrl-baf) for 4 h. Intracellular staining of LC3-II was performed using a two-step staining with anti-human LC3B (4E12; MBL Life Science) followed by goat anti-mouse IgG1 (AF488; Invitrogen A-21121). Stained cells were acquired with FACSymphony A1 flow cytometer (BD Biosciences) and analyzed with FlowJo version 10.6.2.

### Cell lines

The Caco-2 cell line was a gift from the Department of Medical Microbiology of the Amsterdam UMC, location AMC, and was maintained in EMEM (ATCC #30–2003) with 8% fetal calf serum (FCS; Invitrogen), 2 mm L-glutamine (Lonza), 1× non-essential amino acids (SanBio SCC0823), and penicillin/streptomycin (10 U/mL and 10 μg/mL, respectively).

The U87 cell line stably expressing CD4 and wild-type CCR5 co-receptor was obtained through the NIH AIDS Reagent Program, Division of AIDS, NIAID, NIH: U87 CD4^+^CCR5^+^ cells from Deng and Littman [53]. Autophagy reporter cells were generated via retroviral transduction of U87.CD4.CCR5 with pBABE-mCherry-GFP-microtubule-associated protein 1 light chain 3 (LC3) beta (Addgene 22,418; gift from Prof. Jayanta Debnath), as described previously [18,54], hereafter referred to as U87.mCherry-GFP-LC3 cells. U87 cell line was maintained in Iscoves Modified Dulbecco’s Medium (IMDM, Thermo Fischer Scientific, USA) supplemented with 10% FCS and penicillin/streptomycin (10 U/mL and 10 μg/mL, respectively).

### Autophagic flux

U87.mCherry-GFP-LC3 cells were incubated with 5 μM MK-2206, 200 nM Bafilomycin A1 control, or left untreated for 24 h or 48 h. GFP and mCherry fluorescence were measured by multiparameter (FACS LSR-FORTESSA, BD Biosciences) and imaging flow cytometry analysis (ImageStream Mk II, Amnis) as previously described [18,44]. LC3 is an autophagy marker and associates with autophagosomal membranes through lipidation to generate LC3-II. Upon fusion with lysosomes, the inner LC3 is degraded, and while the mCherry signal remains stable, the GFP signal is quenched by the acidic environment. Reduction of the percentage of mCherry puncta simultaneously positive for the GFP signal is therefore representative of autophagic flux and was determined in live, singlet, mCherry^+^GFP^+^ cells. The control gate to determine autophagic flux was set in cells treated with bafilomycin A1[18,44].

### Cell viability

Cell viability was assessed in Caco2 cells or intestinal epithelial monolayers upon treatment with MK2206 for 72 h or 60 h, respectively, using the ATP-based CellTiterGlo 3D reagent (Promega) according to the manufacturer’s instructions. Briefly, the CellTiter-Glo 3D reagent and white 96-well plate were equilibrated at room temperature (RT). Medium was removed from the cells, and 100 μL of 1:1 ratio of CellTiter-Glo 3D reagent : dPBS (Lonza) was added to the cells and mixed vigorously. After incubating for 15 min at 37°C to induce cell lysis, the suspension was transferred to the white 96-well plate, and luminescence was measured by the SynergyHTX (BioTek) plate reader.

### SARS-CoV-2 pseudovirus production and infection

Single-round infection pseudotyped SARS-CoV-2 (NL4.3-ΔENV-luc-GB-pseudo-SARS-CoV-2-SpikeΔ21) was generated as described previously [55]. The SARS-CoV-2 spike plasmid HDM-IDTSpike-fixK-∆21 was obtained through BEI Resources, NIAID, NIH: Vector pHDM Containing the SARS-Related Coronavirus 2, Wuhan-Hu-1 Spike Glycoprotein Gene, D614G Mutant with C-Terminal Deletion, NR-53765. The adjusted HIV-1 backbone plasmid (pNL4–3.Luc.R-S-) containing previously described stabilizing mutations in the capsid protein and a firefly luciferase gene in the *nef* open reading frame was provided by Dr. N. Kootstra (Amsterdam UMC) [56]. Briefly, plasmid amplification was achieved by transformation into DH5α or Stbl3 *E. coli* strain (Invitrogen), respectively, and subsequent co-transfection of plasmids was performed in human embryonic kidney HEK-293T cells using a GeneJuice (Novagen, USA) transfection kit, according to the manufacturer’s instructions. Virus was collected 2 days after transfection and filtered over a 0.2 µM nitrocellulose membrane (Sartorius Stedim, Gottingen, Germany). SARS-CoV-2 pseudovirus titer was determined by luciferase reporter activity after 3 days of infection in HEK-293T cells expressing human ACE2 (HEK-293T-hACE2), obtained through BEI Resources, NIAID, NIH: Human Embryonic Kidney Cells (HEK-293T) Expressing Human Angiotensin-Converting Enzyme 2, HEK-293T-hACE2 Cell Line, NR-52511. Stock titers were determined by titration on Vero cells. The tissue culture infectious dose 50 (TCID50) was calculated according to the method of Spearman & Kärber [57,58]. For infection studies, Caco-2 cells or intestinal epithelial monolayers deemed confluent were treated overnight with 5 μM MK-2206 hydrochloride (Cayman Chemical), 5 nM everolimus (Invivogen), 100 nM sirolimus (Invivogen) or left untreated, before infection with 87.5–350 plaque-forming units (PFU) of NL4.3-ΔENV-luc-GB-pseudo-SARS-CoV-2-SpikeΔ21 for 5 days. SARS-CoV-2 pseudovirus fusogenic entry of intestinal epithelial cells was determined by luciferase activity using a luciferase assay system (Britelite Plus Kit, Perkin Elmer cat #6066761) according to the manufacturer’s instructions.

### RNA interference

RNA interference was performed using the Neon Transfection System according to manufacturer’s protocol (Thermo Fisher). Caco-2 cells were transfected with different short interfering (si) SMARTpool RNAs (Dharmacon), namely siSNAP29 (*M*-011935-00), or siATG13 (*M*-020765-01–0010) or siNon-Target as a control (D-001206-13) at end concentrations of 0.1 µM siRNA per million cells. Transfected cells, as well as non-transfected Caco-2 cells as an additional control, were seeded in 24-well plates in EMEM (ATCC #30–2003) with 8% FCS (Bio-connect), 2 mm L-glutamine (Lonza), and 1× non-essential amino acids (SanBio SCC0823), without antibiotics. After 32 h, adherent cells were washed and incubated overnight either with 5 μM MK-2206 or left untreated. Silencing of expression of target proteins in Caco-2 cells was confirmed by quantitative real-time PCR.

### RNA isolation and quantitative real-time PCRs

mRNA was isolated from Caco-2 cells with mRNA Catcher™ PLUS Purification Kit (Thermofisher), and cDNA was synthesized with a reverse-transcriptase kit (Promega). For real-time PCR analysis, PCR amplification was performed in the presence of SYBR green for 40 cycles in a 7500 Fast Realtime PCR System (ABI). Specific primers for the host genes were designed with Primer Express 2.0 (Applied Biosystems) (Table S1). The cycling threshold (*C*_t_) value is defined as the number of PCR cycles in which the fluorescence signal exceeds the detection threshold value. For Caco-2 cells, expression of target genes was normalized to GAPDH (N_t_ = 2^Ct(GAPDH)–Ct(target)^), and relative mRNA expression in siRNA transfected Caco-2 cells was obtained by setting siNon-Target control to 1.

### Confocal microscopy

Intestinal epithelial monolayers were fixed in 4% paraformaldehyde (PFA; Electron Microscopy Sciences) for at least 30 min at RT, then extensively washed with PBS. Membranes were then cut out of the insert and permeabilized with 0.5% Triton-X100 for 10 min and subsequently blocked with PBS containing 3% bovine serum albumin (BSA) with 0,5% Tween-20 for 1 h at RT, as previously described [18]. Cells were then incubated with the probe Phalloidin CruzFluor^TM^-488 (Santa Cruz Biotechnology) diluted at 1:1500 in PBS containing 3% BSA with 0,5% Tween-20 for 1 h at RT. Nuclei were stained with 300 nM DAPI (Invitrogen). Cells were subsequently mounted in ProLong Diamond Antifade Mountant (Invitrogen). Samples were imaged on a Leica TCSS SP8 X mounted on a Leica DMI6000 and analyzed using LAS X. Quantitative analysis of fluorescence intensity was performed by measuring the mean gray value with the ImageJ software [18].

### Statistical analyses

Data were processed using FlowJo, LLC, version 10 (Treestar), IDEAS version 6.3 (Amnis), and/or GraphPad Prism 9™ (GraphPad Software, Inc.). For paired observations, a two-tailed paired t-test was employed. For unpaired observations, a two-tailed unpaired t-test was employed. Multiple comparisons were analyzed by one-way AN OVA. Relative data were normalized against untreated, virus-infected samples (set as 1), and a one-sample t-test was subsequently used to compare fold changes in experimental conditions to a theoretical mean of 1. All statistical analyses were conducted using GraphPad Prism 9.

## Abbreviations

ATG16L1: Autophagy-related 16 like 1
ATG13: Autophagy-related 13
COVID-19: Coronavirus disease 19
FCS: Fetal calf serum
GFP: Green fluorescent protein
MK-2206: MK-2206 hydrochloride
LC3: Microtubule-associated protein 1A/1B-light chain 3
RLU: Relative light units
SARS-CoV-2: Severe acute respiratory syndrome coronavirus 2
SNAP29: Synaptosomal-associated protein 29
SNP: Single nucleotide polymorphism
TEER: Trans-epithelial electrical resistance

## Supplementary Material

Supplemental Material

## Data Availability

The majority of the data generated or analyzed during this study are included in this article. Requests for data should be made to and will be fulfilled by Dr. C.M.S. Ribeiro (c.m.ribeiro@amsterdamumc.nl), provided the data will be used within the scope of the originally provided informed consent.
