# Supplementary material for: Autophagy-enhancing strategies to promote intestinal viral resistance and mucosal barrier function in SARS-CoV-2 infection
Source: Autophagy Rep. 2025 Jun 10;4(1):2514232. doi: 10.1080/27694127.2025.2514232 (PMC12153388; doi:10.1080/27694127.2025.2514232)
Supplement: Supplemental Material [file KAUO_A_2514232_SM5492.docx]

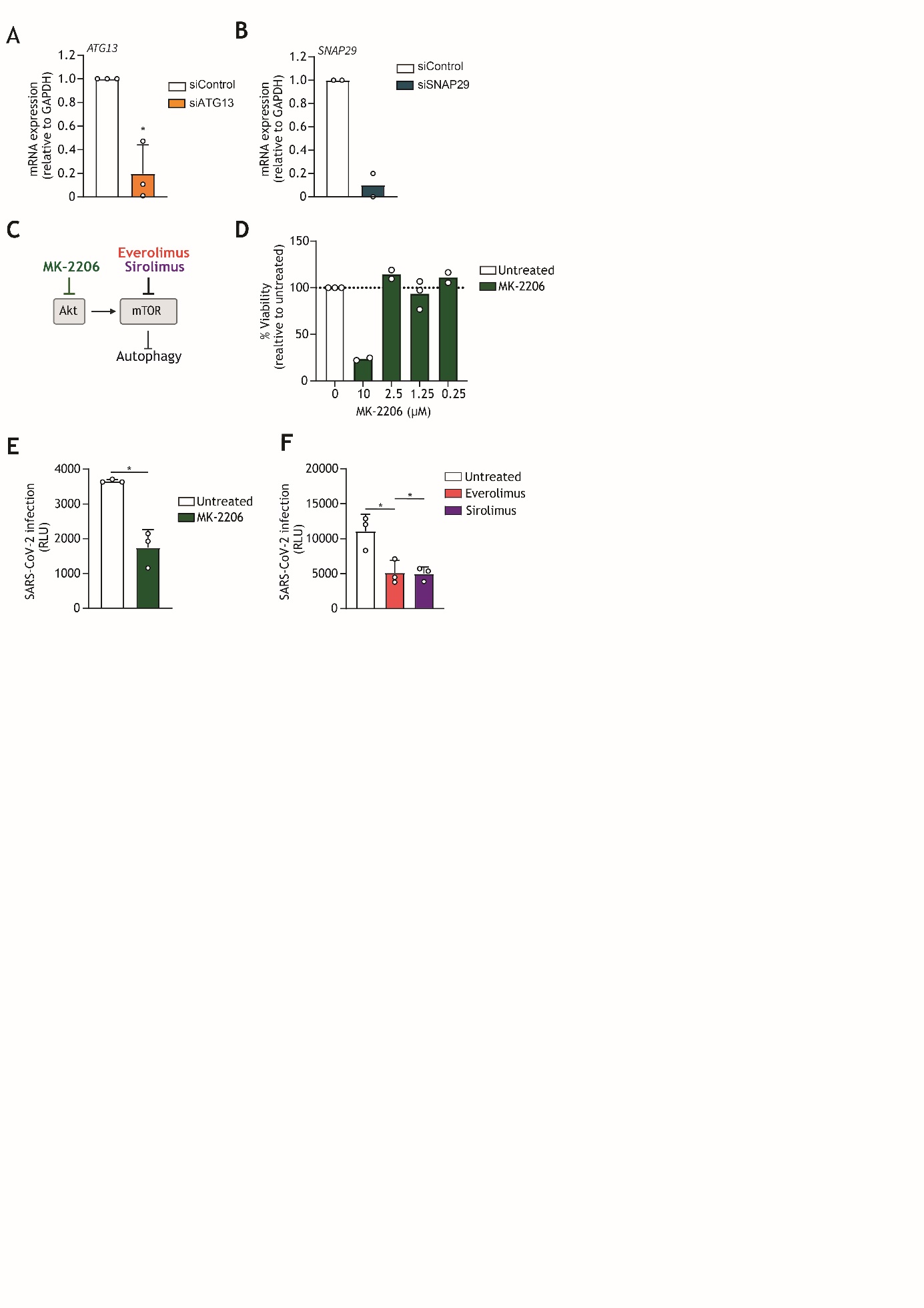


**Supplemental Figure 1.** (**A**) *ATG13* and (**B**) *SNAP29* were silenced in Caco-2 cells using specific SMARTpools and non-targeting siRNA as control. Silencing was confirmed by real-time PCR. mRNA expression was normalized to *GAPDH* and set at 1 in cells treated with control-siRNA. (**A**) *n*= 3 replicates; **P* < 0.05. (**B)** *n*= 2 replicates. **(C)** Schematic representation of the mechanism by which mTOR complex inhibitors Everolimus and Sirolimus impact their molecular target to pharmaceutically increase autophagy flux. (**D**) Percentage cell viability upon treatment of Caco-2 cell line with MK-2206, determined by ATP-based CellTiter-Glo assay. Caco-2 cells were treated with increasing dilutions of MK-2206 or left untreated for 72 h. Open circles represent individual replicates, *n* = 2-3 replicates. (**E**) Viral infection of Caco-2 cells pre-treated with pre-treated with 5 μM MK-2206 or left untreated for 24 h, followed by infection with SARS-CoV-2 pseudovirus for 72 h, determined by luciferase activity (RLU). Open circles represent individual replicates, *n* = 3 replicates; **P* < 0.05. (**F**) Viral infection of Caco-2 cells pre-treated with 5 nM everolimus, 100 nM sirolimus, or left untreated for 24 h, followed by infection with SARS-CoV-2 pseudovirus for 72 h, determined by luciferase activity (RLU). Open circles represent individual replicates, *n* = 3 replicates; **P* < 0.05.

| **Primer** | **Sequence** |
| --- | --- |
| **GAPDH *forward*** | CCATGTTCGTCATGGGTGTG |
| **GAPDH *reverse*** | GGTGCTAAGCAGTTGGTGGTG |
| **ATG13 *forward*** | TGTCCAAGTGATTGTCCAGGC |
| **ATG13 *reverse*** | AACTCCCGATAGAAGGTCCCC |
| **SNAP29 *forward*** | TCATGTACGAGTCCGAGAAGG |
| **SNAP29 *reverse*** | CCCAAACACGCTCTTAATGCTAT |

**Table S1.** mRNA expression primer sequences
